# Participatory Methods in Food Behaviour Research: A Framework Showing Advantages and Disadvantages of Various Methods

**DOI:** 10.3390/foods10020470

**Published:** 2021-02-20

**Authors:** Marleen C. Onwezen, Emily P. Bouwman, Hans C. M. van Trijp

**Affiliations:** 1 Wageningen Economic Research, Wageningen University and Research, 2595 BM The Hague, The Netherlands; emily.bouwman@wur.nl; 2 Marketing and Consumer Behaviour Group, Wageningen University and Research, 6708 PB Wageningen, The Netherlands; hans.vantrijp@wur.nl

**Keywords:** participatory research methods, food consumption, framework

## Abstract

A trend is visible in the food literature showing an increasing number of publications on studies that incorporate some form of participant engagement, such as citizen science and community-based participatory research. This “participation trend” will inevitably affect the scientific field of food behaviour research. This new trend is however not only associated with advantages, and a critical reflection on both the advantages and disadvantages is needed. The current article is a position paper that contributes to the literature in two main ways. First, participation is still in the developmental stage. Many different forms, methods and definitions are used. By providing a structured overview of a variety of participatory methods derived from a focused search of the literature on food behaviour, we aim to clarify the relationships between the various forms of participation methods. Second, the involvement of citizens in research is increasingly calling for novel research methods (e.g., voluntary recruitment and active involvement), which may be accompanied by both advantages and disadvantages. We add to the literature by developing a framework that indicates the advantages and disadvantages of participatory methods in food behaviour research. Our study highlights the relevance of differentiating the goal of the researcher (efficiency versus engagement) and the role of citizens (collecting versus creating), thus implying a trade-off between cost-effectiveness and involvement, as well as between data richness and data quality. Our work is a first effort to create structure and guidance within a new area. Our efforts could be used in future research aimed at developing more extensive protocols and tools for the application of participation in research, thereby offering a controlled manner to ensure that research stays abreast of our changing society.

## 1. Introduction

The food literature now comprises an increasing number of publications on studies that incorporate some form of participant engagement (e.g., citizen science, community-based participatory research, participatory research) in their methods [1,2,3]. A literature search including a wide variety of terms that have been used to refer to participation in research (e.g., citizen science, co-creation, participatory research and crowd science) revealed that the majority of such studies (~70%) had been published in the past five years (see the Results section for additional details on the search string). More specifically, participant engagement involves a process in which individuals actively participate in research processes that affect them personally (e.g., citizen participation, as defined by Florin and Wandersman [4]). Participatory methods encompass a wide range of different methods (e.g., citizen science, community-based participatory research, participatory research). These methods have in common that they all use active and conscious partaking of participants in (parts of) a research process, that the traditional researcher–participant role division becomes less strict and that they together co-design and/or execute (parts of) the research. The participation of respondents is conscious, in that they deliberately and transparently decide to contribute to a specific part of a research (i.e., excluding observatory research or use of secondary data).

From a philosophical perspective, the trend towards more participation reflects a transition from a more positivistic approach towards a more social constructivist approach [5,6]. In other words, the literature is undergoing a transition from a top-down approach, in which the researcher defines all the rules and concepts, towards research designs in which knowledge is built together, in which respondents are being engaged in research in various ways. Research methods have historically been reactive, relying on past experiences and minimising interaction with participants [7,8] in order to ensure the validity and reliability of the results. Participatory methods can be distinguished from these research methods, as they include active and conscious forms of citizen participation in at least part of a research process in which the traditional relationship between the roles of the researcher and the participant role becomes less rigid and in which the researcher and participant jointly co-design and/or execute the research.

The involvement of citizens in research is increasingly calling for novel research methods (e.g., voluntary recruitment and active involvement in the research design), which may be accompanied by both advantages and disadvantages. Possible advantages could include increased efficiency of data-collection [2], increased external validity [9], increased engagement [10], the ability to reach a larger sample [11] and the ability to receive input from a variety of stakeholders [12]. Disadvantages could include less objective measures [13], less representative samples [14], higher costs [12,15,16], decreased reliability [17] and decreased internal validity [7]. Given that participation in food research is an emerging area, critical reflection is needed on this “participation trend” in research on food behaviour [8] (defined as all social sciences within the context of food, thus including food choices, food consumption and food behaviour).

The need for critical reflection on the trend towards participatory methods is based on several factors. First, research on participation is still in the developmental stage. Many different forms, methods and definitions are used (for an overview, see Kujala [12]). For example, the methods and definitions used in this stream of research include citizen science [11], participatory research [18] and community-based participatory research [18]. Although the various forms of participation clearly reflect different methods and/or aims, the relationships between these forms are not entirely clear. In the current study, we provide an overview of the relationships that exist amongst the various methods and definitions. Our scope is restricted to participatory methods that include a research objective as their main focus. In addition to clarifying the similarities and differences existing amongst methods, this overview could allow the various streams of research to build upon each other.

A second reason why critical reflection on participatory methods is needed is that the active engagement of participants has apparently only recently been applied within the context of food (mostly within the past five years^1^). Although its use is more advanced in other areas, including biology (i.e., data monitoring by citizens [19]), some aspects of participation research are apparently more applicable within the context of food than others are. Our study is intended to provide an overview of the advantages and disadvantages of current scientific approaches, based on the literature on participation methods in general, as well as on specific applications of these methods within the context of food. To the best of our knowledge, our manuscript is the first overview of this literature within the context of food. The overview of advantages and disadvantages of various participatory methods is a contribution to the literature.

The current article is a position paper that contributes to the literature by providing a structured overview of a variety of participatory methods derived from a focused search of the research literature on food behaviour (Section 2) and by developing a framework that indicates the advantages and disadvantages of participatory methods in food behaviour research, as derived from our analysis of cases from both the food literature and the general literature (Section 3). We identify two dimensions, using them to develop a structured overview of definitions of participatory methods, along with their advantages and disadvantages. We conclude the article by reflecting on the current and future use of participatory methods in food behaviour research.

## 2. Participatory Research Methods and Applications in Food Behaviour Research

### 2.1. A Structured Verview

The objective of this study is to provide an overview of the most relevant forms of participatory methods applied in food research and to identify underlying dimensions in order to develop a structured overview of the various forms of participation. We consulted existing reviews [1,2,3,11,14] to develop the following search string aimed to find a wide range of studies that use participatory methods in food behaviour research: (LANGUAGE (english) AND DOCTYPE (ar OR ip OR bz OR cp) AND (TITLE-ABS-KEY (“social innovation” OR “paticipatory research” OR “participation research” OR “participation study” OR “participation science” OR “participation method” OR “co-creation” OR cocreation OR “co-producers” OR coproducers OR “co-creators” OR cocreators OR “co-implementer” OR “co-designer” OR codesigner OR “citizen science” OR “community science” OR “crowd science” OR “crowdsourcing” OR “curriculum-based projects” OR “voluntary biological monitoring” OR “action-based research” OR “reversed design” OR “community-based participatory research” OR “community-based monitoring” OR “community-based management” OR “community-based participatory approach” OR “community-based participatory intervention” OR “civic engagement” OR “participatory photovoice” OR “youth participatory action research”)) AND (TITLE-ABS-KEY (food OR dish OR “food consumption” OR “healthy diet” OR “sustainable diet” OR “healthy consumption” OR "sustainable consumption” OR “healthy nutrition” OR “sustainable nutrition”))) including a large variety of broad terms that have been used to refer to participation in research (e.g., citizen science, participatory research and crowd science). 

On 7 August 2019, a Scopus search using this string yielded 956 hits, which is equivalent to ~0.1% of the total food behaviour literature. This percentage is derived from hits resulting from the same search string excluding only the terms referring to participation in research (i.e., only food literature). Of these hits, the majority had been published in the last five years (~70%), indicating that participation in food research is a recent, growing trend. A title scan of the most cited articles yielded the current overview of various participatory methods in food behaviour research.

We do not claim that our overview is comprehensive, given the complexity and number of participatory methods. For example, several terms have been used to refer to citizen science (e.g., community science), and the concept has been used with reference to the process of community-based monitoring and/or community-based management, which is also known as voluntary biological monitoring [11]. Another example is participatory action research, which has also been referred to with a variety of terms (e.g., “collaborative action research” and “action research”). Participatory action research is similar to the original version of action research, but with greater emphasis on participation, even though action research is participatory by definition (see McNiff and Whitehead [20], for more examples).

The four most relevant streams of participatory methods that have been included in this overview are: citizen science, crowd science and two methods that are derived from participatory action research, namely photovoice and community-based participatory research (see Table 1). Given our focus on research methods, we did not include social innovation [3], which is more a concept than a method, nor co-creation [3,21] or circular food design [22], which focuses more on product development, or hybrid forum [23], which focusses more on supporting a dialogue towards developing a democratic world. We thus focus on participatory methods that have a main focus on research or increasing scientific insights on food behaviour.

### 2.2. Dimensions of Participation in Food Behaviour Research

**Figure 1:**
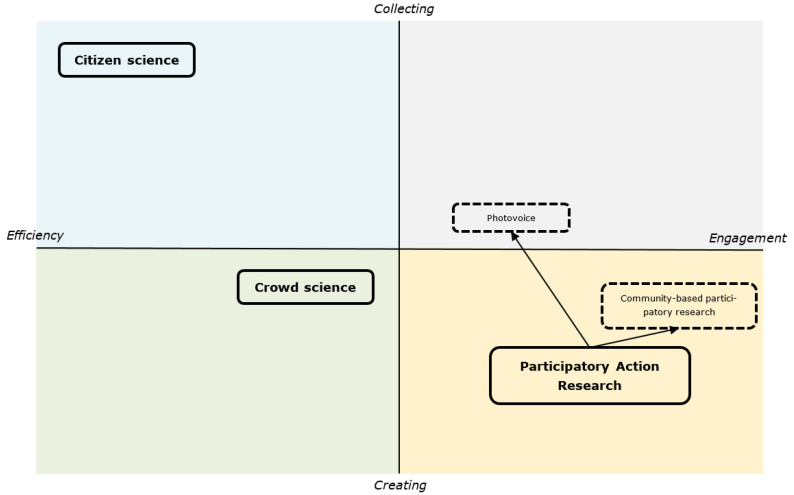
Dimensions of participation methods in food behaviour research.

We developed a framework to provide a structured overview on how the various participatory methods relate to each other. From the perspective of the researcher, we identified two dimensions that categorise the broad range of forms of participation used in food behaviour research. First, the goal of a researcher could be to involve citizens for reasons of either (1) efficiency (e.g., in terms of cost and time) or (2) engagement (e.g., to reduce the gap between citizens and science). Second, the specific role that citizens may receive from a researcher could involve either (1) collecting (e.g., citizens as data collectors) or (2) creating (e.g., citizens as research assistants helping to set up a study). Note that both of these dimensions imply a trade-off that researchers must make when deciding to involve citizens. In some cases, a researcher might have multiple goals and a citizen might have multiple roles, although such instances always reflect a clear prioritisation of the aforementioned dimensions. The participatory methods were allocated into the dimensions according to where the greatest emphasis lies. The categorisation of the various terms into the two dimensions is presented in Figure 1. 

#### 2.2.1. Quadrant 1: Efficiency and Collecting

Participation in research was originally developed in response to a lack of data, or in response to inadequate or incomplete data [11]. This was followed by the development of citizen science, a process in which citizens (i.e., amateurs or non-professionals) are actively involved in science as data collectors, often as volunteers [11]. Citizen science is an example of a participation strategy that focuses primarily on increasing ‘Efficiency’ in research by asking citizens to collect data. Citizens are asked to collect or provide information in a specified manner. Their role is thus that of the “worker”, with their tasks being specified within the investigator’s research design.

Citizen science is used predominantly in the bio-sciences, as in the monitoring of water or air quality, animal populations (e.g., birds, amphibians and fish), and plants [11]. Citizen science has nevertheless been applied in food behaviour research as well. For example, the “food profiler” is a smartphone application in which citizens can track what they eat [24]. The food profiler provides a platform through which citizens can track their own consumption patterns, thereby contributing to research. Another example of a smartphone application that can collect citizen data is “Dyet” (Do you eat this? [26]), a gamified app devised for collecting data on additives in food products and for informing app users concerning the presence of such additives in commercially available food products. The goal of Dyet is to build a database of harmful additives and products containing them, also in addition to raising awareness about food additives amongst the general public.

#### 2.2.2. Quadrant II: Efficiency and Creating

While the development of the internet has greatly facilitated citizen science projects [27], it has also enabled an entirely new category of citizen science methods focused on data processing: crowd science. Crowd science is a scientific research method that is supported by a crowd, making otherwise unfeasible projects feasible (e.g., data-rich or labour-intensive projects). Based on the literature we state, the main goal of researchers implementing crowd science is ‘Efficiency’. Given that crowd science draws on a large number of volunteers through online recruitment, however, ‘Engagement’ seems to play a role as well, albeit a secondary one [2]. Volunteers participate independently of time and place and through gamification or other novel methods [2]. Crowd science appears to complement rather than replace the role of the professional scientist [28]. It can be distinguished from citizen science, as it is more scalable and citizens are involved in multiple steps of the research, not just in data collection. Volunteers thus have a ‘Creative’ role as well, which appears to receive slightly more emphasis than the ‘Collecting’ role does in crowd science. One example of crowd science is the Quantified Self movement (http://quantifiedself.com), which motivates citizens to participate in science by tracking themselves, asking questions and sharing knowledge. Topics focus primarily on health, of which food and nutrition are a part. Another emerging form of participatory research in this quadrant is crowd-sourced research, in which professional researchers actively ask for resources (e.g., money, time or data) from the general public in order to perform their research.

#### 2.2.3. Quadrant III: Engagement and Collecting

One participation method that fits in this quadrant is photovoice, which was derived from participatory action research (PAR, see Quadrant IV) and which is defined as “a process by which people can identify, represent, and enhance their community through a specific photographic technique” [10,29]. In photovoice projects, citizens take photographs of their environment, which then serve as the data for the study. Although photovoice techniques are used to ‘Engage’ a community, the role of citizens focuses more on ‘Collecting’ than ‘Creating’. This form of participatory research is thus classified in the third quadrant.

One example of a photovoice project in food research is a study by Díez and colleagues [10], who asked participants to take photos of their food environments, in order to generate insight into the characteristics of the local food environment within a low-income urban area that influence the diets of area residents. Another example is a study by Belon, Nieuwendyk, Vallianatos and Nykiforuk [30], who asked participants from four communities to take pictures of barriers to and opportunities for healthy eating and to share their stories in interviews.

#### 2.2.4. Quadrant IV: Engagement and Creating

Participatory action research (PAR) is a collaborative process of research, education and action. In PAR, researchers and participants work together to examine a problem and to find ways to solve it [18]. This form of participatory research to ‘Engage’ citizens directly in the research process, and the main role of citizens is to ‘Create’. It is based on action research [20], and both of these participatory strategies share common principles [31,32]. Given that PAR tends to be used to emphasise the participative nature of action research [20], we decided to include PAR in our framework. A variety of different research methods can be linked to PAR, including community-based participatory research [18].

Community-based participatory research (CBPR) is a method in which researchers co-conduct research with citizens from a community and in which community members are involved in the entire research process. This approach bridges the gap between knowledge produced through research and what is already being practiced in communities [33]. This form of participatory research ultimately leads to a deeper understanding of a community’s unique circumstances, and it has been said to offer a more accurate framework for testing and adapting best practices to the community’s needs [33]. The method is often used when the researcher’s goal is to ‘Engage’ citizens and to allow citizens to have both a ‘Collecting’ role and a ‘Creating’ role, although there is more emphasis on the creating role. For example, two studies involved the formation of advisory boards with children from the community in order to obtain feedback on the design, development and implementation of a food literacy programme [25] or to improve the quality of the food served in a school cafeteria [34]. The contributions that the students made to the research included defining the problem, designing the methods to collect data, collecting and analysing the data, and presenting the findings. Another example is a study that involved conducting an inventory of selected markets in areas with high and low concentrations of African Americans [13]. Citizen activities included attending an advisory group, finalising measurement instruments, hiring students to profile the nutritional resource environment and sharing data with study participants, community residents and policymakers.

Although the body of evidence on participation in research on food behaviour is relatively modest, it thus covers a wide range of ways in which participation is implemented. These forms of participation differ according to the goal of the researcher and role of the citizen.

## 3. Applying Participation in Food Behaviour Research: Advantages and Disadvantages

We address two different angles to obtain a comprehensive overview of advantages and disadvantages: the general literature and the food behaviour research literature.

**Figure 2:**
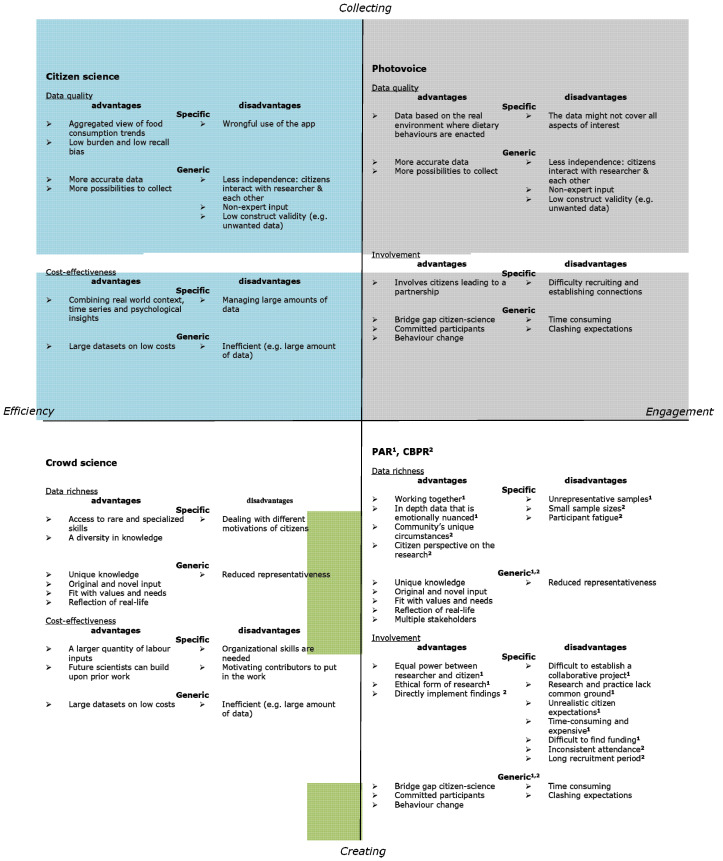
Most substantial advantages and disadvantages per dimension, participation method and area from food behaviour research and the general literature. NB. Not all pros and cons are named here. Only the most important ones. See Appendix for a detailed overview from the food literature.^1^ refers to advantages and disadvantages for PAR (PAR = Participatory action research); ^2^ refers to advantages and disadvantages for CBPR (CBPR = community-based participatory research).

First, given that researchers might be biased in stating the advantages and disadvantages of their research, we start with a description of the general literature in order to develop an overview of the advantages and disadvantages of various forms of participatory methods, as compared to methods that do not involve participation. Second, we describe specific advantages and disadvantages that have been mentioned in the food behaviour research in relation to each of the quadrants outlined in Section 2: (I) efficiency and collecting; (II) efficiency and creating; (III) engagement and collecting; (IV) engagement and creating. Third, the advantages and disadvantages are linked to our developed framework (Figure 2), such that we visualise how the various participatory methods relate to the specified advantages and disadvantages. For the food-specific (dis)advantages the link is based on the literature, for the general (dis)advantages the link is based on interpretation of the authors.

The overview includes a wide range of advantages and disadvantages. Given our overall aim of identifying consistencies across the various participatory methods, we defined four categories of advantages and disadvantages, based on the dimensions outlined in Section 2: cost-effectiveness, involvement, data richness and data quality.

### 3.1. An Overview of Advantages and Disadvantages from the General Literature

Based on the general literature and the dimensions outlined in Section 2, we identify four areas of advantages and disadvantages.

#### 3.1.1. Category 1: Cost-Effectiveness

*Advantages.* Cost-effectiveness is an important motivation for decision-making with regard to research designs [15,16]. Involving citizens as data collectors can be very efficient, and it may generate large datasets at relatively low costs (e.g., citizen science [11]; crowd science, [2]). For example, with smartphone applications, citizens can collect personal data (e.g., on consumption, GPS, and number of steps walked) for long periods of time. These methods are especially well-suited for research questions that can be answered only with large amounts of data, the collection of which can be expensive and time-consuming (e.g., consumption patterns or emotions at various moments, or large scale studies such as including context in segmentation studies [35]).

*Disadvantages.* Participation in research can also decrease efficiency. For example, it can generate an overwhelming amount of raw and unstructured data [12]. Cost-effectiveness is an important criterion for decision-making with regard to research designs [15,16], and some research questions do not require large or specific data sets. In these cases, it might be more efficient to use data-collection methods that do not involve citizen participation.

#### 3.1.2. Category 2: Involvement

*Advantages*. Involving citizens in research can enhance a sense of engagement amongst citizens, potentially bridging the gap between science and practice. By involving citizens, researchers can gain specific knowledge and increase their awareness of scientific and societal issues [2]. Engaged citizens can also enhance participant commitment, thus potentially stimulating long-term behaviour change [36]. Given that individuals determine who they are in part according to their own behaviour, active involvement in research has the potential to influence both attitudes and behaviour (e.g., self-perception theory [37]; cognitive dissonance theory [38]).

*Disadvantages*. The main disadvantage of citizen involvement is that interaction with citizens can be time-consuming [12]. The time spent on a study and on communication with citizens can be seen as an obstacle to citizen involvement. Moreover, the effort that citizens put into a study is not always what the researcher asked for and, conversely, the expectations that citizens have with regard to being involved in a research project are not always realistic [12].

#### 3.1.3. Category 3: Data Richness

*Advantages.* The involvement of citizens in research could potentially enhance the possibility of receiving valuable input on various aspects of the research (e.g., crowd science [28]). For example, citizens may possess unique cultural or societal knowledge, local knowledge and extensive social networks [39]. Asking citizens or respondents to participate in thought processes and to provide input might result in original ideas [8], novel insights, richer input [2,12] and a better fit between the research and the underlying values and needs of citizens [7,8]. Moreover, participatory methods allow for the inclusion of a diversity of stakeholders, such as consumer organisations, industry, farming organisations, civil society, NGOs, and policy makers [12,40]. Overall, the findings of participatory research can provide a better reflection of real life, as citizens are capable of providing more authentic and unique experiences that would not be gathered through other methods.

*Disadvantages.* The downside of insights from a specific community is that the use of a specific sample can reduce the representativeness of the data. In general, researchers strive to study representative target populations. Asking citizens to participate in a study on a voluntary basis decreases the representativeness of the sample [41]. This is a frequently mentioned disadvantage of participation research [16]. For example, due to selection bias [42], only specific groups of individuals will be interested in joining a study voluntarily. In other words, only motivated individuals are interested in participating in research, thus making it more difficult to generalise the findings to other populations or settings.

#### 3.1.4. Category 4: Data Quality

*Advantages.* The primary goal of scientific research is to find answers to specific research questions in an independent manner, with the aim of obtaining reliable and valid outcomes. Despite the difficulty of ensuring such outcomes when citizens participate in research, some aspects of participation can lead to high-quality data. For example, using smartphone applications in data collection and letting citizens help can make it possible to collect data that are more accurate, and thus of higher quality [12]. It can also increase response rates and improve the ability to capture the true meaning of concepts by allowing for more diverse input.

*Disadvantages.* The involvement of citizens in research designs can jeopardise the independence of the researchers, as well as the validity (e.g., does the research measure what it aims to measure?) and reliability (e.g., is the method consistent?) of the results. For example, it might be difficult to hide the research question from respondents, which could affect their response strategies, due to a preference for giving socially desirable answers [43]. Alternatively, researchers could be influenced by the respondents, thus compromising their ability to test hypotheses that have been formulated in advance (research integrity), or respondents could influence each other, thus potentially affecting their response strategies as well. Moreover, citizens are not always experts on the subject being investigated, and their input might therefore decrease the quality of the data. Finally, the involvement of citizens in research requires the researcher to relinquish a certain amount of control with regard to the study [7,17]. For example, citizens might request changes to the study [12] or provide data that go beyond the research question, thus potentially decreasing construct validity. This raises questions concerning the extent to which it is clear what exactly is being measured. For this reason, it is important to develop procedures that leave as little confusion as possible regarding the scope of a participation exercise and its expected output [16,44].

### 3.2. An Overview of Specific Advantages and Disadvantages Mentioned in the Food Behaviour Research, for Each of the Quadrants

In this section, we elaborate on the advantages and disadvantages that are mentioned in food behaviour studies with regard to particular participatory methods. A full overview of the advantages and disadvantages is presented in Appendix A (Table A1). In the text, we focus on the most important advantages and disadvantages for each quadrant, based on consistencies across studies and the general literature.

#### 3.2.1. Efficiency and Collecting (Citizen Science)

The main advantages of participatory methods in this quadrant of citizen science are increased cost-effectiveness and data quality. Large groups of citizens can be reached more easily within the context of real-life decision-making. For example, the food profiler [24] and Dyet [26] are examples of smartphone applications that can be used to collect data on food (and food consumption) for research purposes. The main advantage of the food profiler is that it combines a real-world context, time series and socio-psychological insight by following citizens with an app (cost-effectiveness). Moreover, it uses multiple measurement moments with two-hour recalls and random intervals, thus resulting in a low burden for citizens (data quality) and a low level of recall bias (data quality), thereby enhancing the accuracy of the data. This method can generate an aggregated view of food-consumption trends (data quality), thus generating additional insight for the researcher [24].

Disadvantages of participatory methods in this quadrant include the difficulty, time and costs associated with keeping participants involved. One challenge of using a smartphone application for citizen data collection is that the citizens must be encouraged to use the application properly in order to experience the full benefits of the tool (data quality). They must also be (and stay) motivated to use or start using the tool (involvement). Moreover, such applications generate large amounts of data, which must be managed (cost-effectiveness). According to a literature review on the use of smartphone applications to promote healthy diets, volunteers prefer applications that are quick and easy to administer and that increase awareness of food intake and weight management [14].

#### 3.2.2. Efficiency and Creating (Crowd Science)

Advantages and disadvantages of crowd science can be derived from two factors that distinguish crowd science from other research approaches: open participation and the open sharing of intermediate inputs [45]. Advantages of open participation include a larger quantity of labour input (cost-effectiveness), access to rare and specialised skills (data richness) and knowledge diversity (data richness). One advantage of sharing intermediate inputs is that it allows future scientists to build on previous work (cost-effectiveness) [45].

One disadvantage associated with crowd science is that the large number of projects and contributors makes it difficult to integrate contributions (cost-effectiveness). Another disadvantage concerns how to deal with potential differences in the motivations of contributors (data richness) [45].

#### 3.2.3. Engagement and Collecting (Photovoice)

Photovoice involves citizens in research projects (involvement), which can lead to a partnership (involvement) [10]. This method can generate a high-quality dataset that reveals the actual, experienced environment in which dietary behaviours are enacted (data quality) [30]. One disadvantage is related to the difficulty of establishing community connections and recruiting volunteers (involvement) [10]. It can also be difficult to explain to citizens exactly what the purpose of the study is, and exactly which data are to be collected. If this is not clear, the data that are actually collected might not cover all aspects of interest (data quality [30]). For example, citizens might record only micro-environment attributes and not macro-environment attributes [30].

#### 3.2.4. Engagement and Creating (PAR)

All forms of Participatory Action Research (PAR) [9] assume equal power relations between the researcher and the participant (involvement), with the goal of jointly addressing problems, generating knowledge and finding solutions (data richness). In this process, researchers also give something back to the participant, rather than simply taking up time and energy and giving little in return. Aiken [9] argues that PAR is therefore more ethical (involvement) than other research methods that do not involve participation. Moreover, being part of the process can lead citizens to open up more, thus possibly generating data that are richer, more in-depth, more substantial and more emotionally nuanced (data richness).

One disadvantage of PAR is that it can be difficult to establish a genuinely collaborative project (involvement) [9]. In some cases, it is not possible to identify any common ground between research and practice. In other cases, participants might have unrealistic expectations concerning what the research can achieve for them (involvement). Moreover, PAR tends to be a time-consuming method given the need to train participants, and it can be expensive to keep respondents involved (involvement). It can also be difficult to obtain funding for PAR projects (involvement), as the research questions and hypotheses emerge during the research process, while funders often require hypotheses and research questions to be formulated in advance. Finally, the samples of participants in PAR are often non-representative (data richness) [9].

Community-based participatory research (CBPR [18]) is a method that can be linked to PAR. One advantage of CBPR is that it ultimately generates a deeper understanding of the unique circumstances of a community, along with a more accurate framework for testing and adapting best practices to the needs of the community (data richness) [25,33,34]. The unique perspectives of community residents on research problems and processes is valuable (data richness) [13]. Moreover, the applicability of the research findings to specific communities makes them particularly well-suited for putting results into practice directly within communities (involvement) [13]. Along with its advantages, however, CBPR is subject to a number of disadvantages. Based on their application of CBPR in an adolescent community, Wickham and Carbone [25] identify limitations including small sample size (data richness), long recruitment periods (involvement), inconsistent attendance at pilot program sessions (involvement) and participants becoming fatigued (data richness).

#### 3.2.5. Overview of Advantages and Disadvantages Relating to Method, Quadrant and Area

Drawing on all of the information gathered on the advantages and disadvantages (from both general and food-specific literature), we provide an overview of the advantages and disadvantages for each quadrant (see Figure 2). Although advantages are displayed in all quadrants, data quality is associated with more disadvantages than advantages. Moreover, the results reveal associations between the advantages and disadvantages, such that decisions concerning the various types of participatory methods apparently call for trade-offs. A choice for involvement might decrease cost-effectiveness, and a choice for data richness might decrease data quality (e.g., reliability).

## 4. Discussion

As demonstrated by the results of our study, the body of research on participation in the domain of food behaviour is growing, and researchers are using a variety of methods and definitions. In this position paper, we present a framework for categorising the various methods and definitions, as well as for clarifying how they relate to each other. We categorize the methods and definitions into two aggregated dimensions: the goal of the researcher (efficiency versus engagement) and the role of the citizen (collecting versus creating). Additionally, we used the developed framework to map the various advantages and disadvantages associated with applying participation in food behaviour research. The results reveal that a choice for a specific participatory method necessarily involves a trade-off between four categories of advantages and disadvantages: cost-effectiveness, involvement, data richness and data quality. The creating role of citizens is especially linked to advantages in terms of data richness, whereas the collecting role of citizens is especially linked to data quality. These advantages are also associated with disadvantages, and they require researchers to engage in balanced reflection on their goals. A choice for involvement might decrease cost-effectiveness, and a choice for data richness might decrease data quality (e.g., reliability).

### 4.1. Making a Balanced Choice

This article represents a first attempt to simplify the complex field of participatory methods. Our argument is not that participatory methods should be used in all types of research. Instead, we argue that the use of participatory methods is justified only if the advantages outweigh the disadvantages. As demonstrated by the overview of advantages and disadvantages, such trade-offs differ across the various forms of participatory methods. Researchers could use the overview to make balanced choices concerning the appropriateness of using participatory methods for their own research designs. Despite the utility of our overview, it is important to consider several issues associated with it.

First, the advantages and disadvantages formulated in our overview could be influenced by the level of citizen involvement in a given research project. Blackstock, Kelly and Horsey [46] distinguish five levels of citizen-researcher involvement: informing, consulting, co-deciding, delegating and supporting. Higher involvement is associated with a higher level of engagement [36], but also with less control over the study, and all of the associated disadvantages. Similarly, lower involvement implies greater control for the researcher, albeit potentially at the expense of citizen engagement. This variable of “control” can be seen as an additional dimension of the framework, which can strengthen both the advantages and the disadvantages.

A second issue with our overview is that it cannot be seen as a fixed output. A researcher can influence both the advantages and the disadvantages. Issues of reliability, validity and other aspects can be addressed and, to a certain degree, resolved by gaining control over the level of involvement at the proper time. For example, validity can be improved when researchers make the ultimate decisions themselves, give clear instructions to the data-gatherers or set certain limits on the additional information that respondents can provide. Technological innovations can provide opportunities for reaching a broad, representative range of citizens who could be involved in a study. Moreover, other research designs (e.g., randomised controlled trials) can help to confirm that the findings of a specific intervention are truly the result of the intervention.

Third, it is important to note that different participatory methods can be used in different phases of research. At an aggregated level, a research trajectory can be divided into the following processes: initiation, development and assessment [47]. We propose that each research phase calls for a different role on the part of participating citizens: a creating role (initiation), a working role (development) and a reflecting role (assessment). As defined in our dimensions, the creating role is relevant at the start of a study (e.g., for determining the research question) and at the end of a study (e.g., for reflecting on conclusions and implications), whereas the working role (defined in our dimensions as “collecting”) is relevant in the middle of a study, as participants are being recruited (as sources and collectors of data), the study is being implemented and the data are being gathered and analysed. The trade-offs that researchers must make are thus likely to differ according to the stage of research to which participatory methods are applied.

A fourth issue with the overview is a focus on immediate implications from research. Nevertheless, the use of participation in different phases of a study might also have long-term consequences for researchers and citizens. In the long run, it might be possible to close the gap between research and society by establishing closer interaction and a more balanced relationship. Enhanced mutual understanding of why certain choices must be made could lead researchers to recognise the value of various forms of input more and citizens to value the scientific approaches more. This could be instrumental in decreasing gap between science and society [48]. Another example of long-term consequences might involve the difficulty of replicating research findings when the samples on which they are based are not representative. This could have consequences for the body of research on food consumption behaviour in terms of trust in science, the ability to build on previous research and the explanation of contradicting findings.

### 4.2. Current Use of Participatory Methods in Food Behaviour Research

The current study indicates that the use of participatory methods within the domain of food behaviour science is lagging behind other domains. In other areas (e.g., commercial research and policy-making), citizens are becoming increasingly empowered and engaged in activities to ensure that the processes and outcomes of food developments are in line with their needs and preferences [3,16,21,49,50]. In this section, we reflect on possible reasons for this trend, based on the advantages and disadvantages identified in this study.

First, the engagement of participants is apparently a less prominent goal in food behaviour research as it is in commercial research and policy-making. In commercial research and policy-making, engaging citizens is closely associated with the ultimate goal of an end product: acceptance by citizens or consumers (e.g., the purchase of products or the acceptance of policy measures). In contrast, the goal of scientific research is to find answers to a broad range of questions in a less instrumental manner (e.g., “what are current food patterns?” or “which mechanisms drive the acceptance of new proteins?”). Although participant engagement can be a secondary goal for a study (e.g., in order to decrease the gap between science and society), this goal does not overlap with the research goal to the same extent as it does in commercial research and policy development. This is because, by definition, the outcomes of scientific research are not known in advance, and the engagement of end users is not the main purpose of conducting scientific research [18].

A second reason why food behaviour scientists have not yet widely embraced participant engagement could be the level of expertise needed for research in this field (in contrast to commercial research and policy-making). Scientific research has historically proceeded from the assumption that the comprehension of complex decisions should best be left to experts and scientists, as citizens likely to be regarded as having limited capabilities and deficient knowledge [16]. Scientific research calls for a different level of expertise from citizens than is needed for commercial research and policy-making. In the development of policies and products, citizens are regarded as experts in expressing preferences and, therefore, an excellent source of information for purposes of developing the right product or cultivating acceptance of particular products or policy measures. In food behaviour research, participants are often not experts, as such studies often focus on complex, abstract or unconscious processes of decision-making (e.g., unconscious decision-making or the role of social norms). For this reason, lay input is not always of added value.

Finally, the relative difficulty of measuring food behaviour could also help to explain the lack of widespread application of participation within this field. Participatory methods appear to be an emerging trend in food science, whereas they are already more anchored in other domains of scientific research (see citizen science in biology [19]). The measurement of specific food choices calls for objective, detailed measures. In addition, unravelling the underlying processes goes beyond rational decision-making to include unconscious and affective processes of which citizens are unaware. The relative complexity of the specific context of food behaviour research might therefore make it difficult to integrate participatory research methods.

### 4.3. Participatory Methods in Future Research: A Research Agenda

As indicated by our overview, participation is used in various ways in the food literature. Our study represents a first effort to reveal the relationships between the various forms of participation in research. The results highlight the need for clarification and simplification with regard to the definition of participatory methods. This could greatly enhance the literature, as it would allow researchers to build on each other’s work. The lack of—and need for—vested methods and theory has also been mentioned within the context of participation in public policy debates [51]. Future research should develop an integrated conceptual model to help researchers from various domains to link their efforts to the body of literature.

A related issue is the tools that are available to researchers for the application of participatory methods. These tools vary across and within specific participatory methods. The development of protocols and tools for applying participatory methods could greatly enhance the quality and ease of their use in research. Moreover, it would be highly useful to use similar methods in terms of comparisons across studies [52].

As demonstrated in the current study, although several different participatory techniques have been developed, the effectiveness of the various methods of participation has yet to be compared systematically. In this article, we present a framework of advantages and disadvantages that could also be used to provide criteria for evaluation. It can also be used to guide a preliminary assessment of the effectiveness (e.g., data quality, involvement, long-term behaviour change) of various participatory methods. Future research is needed in order to identify when and under which circumstances various types of participation are most effective.

Novel technologies could potentially enhance the development and use of participatory methods. For example, mobile applications make it possible to reach increasing numbers of people, and sophisticated software could potentially offer a variety of options for linking various groups of individuals to researchers (e.g., through online chats for the creation of living labs). Moreover, increasing opportunities for sophisticated data analysis are being developed. For example, open access statistical programmes (e.g., R) are constantly creating opportunities for analysing unstructured data or linking various datasets. Apps and wearables could allow the food literature to profit from novel technologies that provide near-time access to behavioural choices and that offer various novel research opportunities. For example, such technologies could be used to ask citizens to share their data from various sources (e.g., smartwatch, calorie tracker and activity tracker).

The selection of dimensions, the allocation of participatory methods and the identification of advantages and disadvantages all entail an element of subjectivity. Although our categorisations are intended to structure and guide the current body of research, different dimensions or categorisations might have generated different findings. Future research is needed in order to quantify these results (e.g., by conducting direct comparisons across participatory methods).

### 4.4. Conclusions

Increasing the involvement of citizens in food behaviour research has both advantages and disadvantages. In response to current societal trends, food behaviour studies are increasingly incorporating more extensive involvement of citizens or participants. To date, however, a clear overview of the relationships between the various forms of participation and the advantages and disadvantages associated with them was missing. Our study highlights the relevance of differentiating the goal of the researcher (efficiency versus engagement) and the role of citizens (collecting versus creating), thus implying a trade-off between cost-effectiveness and involvement, as well as between data richness and data quality. Our work is a first effort to create structure and guidance within a new area. Our efforts could be used in future research aimed at developing more extensive protocols and tools for the application of participation in research, thereby offering a controlled manner to ensure that research stays abreast of our changing society.

## Figures and Tables

**Table 1:** The four selected forms of participatory research including definitions and used methods in food behaviour research based on the literature overview.

Selected Forms of Participatory Research	Definition	Main Methods Used in Food Behaviour Research	Illustration
Citizen science	A process in which citizens (i.e., amateurs or non-professionals) are actively involved in science as data collectors, often as volunteers	- Smartphone application - (Online) survey	The “food profiler” is a smartphone application in which citizens can track what they eat [24]. It provides a platform through which citizens can track their own consumption patterns, thereby contributing to research.
Crowd science	Collaborative contributions of a large group of people to the different steps of the research process.	- Internet - Online communication - Self-trackers - Crowd sourcing (e.g., all means to receive valuable input on research tasks, leaving space for creative input)	The Quantified Self movement (http://quantifiedself.com), which motivates citizens to participate in science by tracking themselves, asking questions and sharing knowledge.
Photovoice	A process by which people can identify, represent, and enhance their community through a specific photographic technique	- Taking photos - Focus groups (discussing the photos) - Interviews (sharing stories with photos)	Diez and colleagues [10] conducted a photovoice project with adult residents of a low-income urban area to understand the characteristics of the local food environment influencing residents’ diets. The method involved citizens taking pictures of all the features related to the food environment in the neighborhood.
Community-based participatory research	A method in which researchers co-conduct research with citizens from a community and in which community members are involved in the entire research process.	- Advisory boards (e.g., defining the problem, designing the methods to collect data, collecting and analysing the data, and presenting the findings)	Wickham and Carbone [25] formed an advisory group (Kid Council) to direct the design of a food literacy program and to implement a pilot version of the program to assess participants’ attitudes to participate. Adolescents from the community sat in an advisory group and provided feedback on the program’s design and development.

## Data Availability

Position paper based on literature overview, no primary data was used.

## Appendix A

**Table A1:** Overview of the Advantages and Disadvantages of Using Participation in Food Behaviour Research.

Dimension	Participation Strategy	Advantages	Disadvantages
Efficiency and collecting	Citizen science	Cost-effectiveness ⮚ Combining real world context, time series and socio-psychological insights in one application [1,2,24] Data quality ⮚ A low burden for citizens [24] ⮚ A low recall bias [24] ⮚ An aggregated view of food consumption trends [24]	Cost-effectiveness ⮚ Managing large amounts of data [24] Data quality ⮚ Wrongful app-use, reducing data quality [14] Data richness ⮚ Reduced representativeness due to attracting highly motivated citizens [14] Involvement ⮚ Citizens need to be motivated to use the tool (correctly) [14]
Efficiency and creating	Crowd science	Cost-effectiveness ⮚ A larger quantity of labour inputs [45] ⮚ The opportunity for future scientists to build upon prior work [45] Data richness ⮚ Access to rare and specialized skills [45] ⮚ A diversity in knowledge [45] Involvement ⮚ Being part of a community and the opportunity to contribute to science [45] Data quality ⮚ Internal verification of the results [45]	Cost-effectiveness ⮚ Integrating contributions is challenging [45] Data richness ⮚ Dealing with different motivations contributors might have [45] Involvement ⮚ The need for organizational methods to match projects and contributors and dividing tasks ⮚ Motivating contributors to increase the work they put in [45]
Engagement and collecting	Photovoice	Involvement ⮚ Involves citizens leading to a partnership [10] Data quality ⮚ High quality dataset revealing the real and experienced environment where dietary behaviours are enacted [30] Data richness ⮚ Gaining first-hand knowledge with interpretations that represent community perspectives [10] Cost-effectiveness ⮚ Gaining a partnership that can facilitate the development of future interventions [10]	Involvement ⮚ Establishing community connections and recruiting volunteers is difficult [10] Data quality ⮚ The data might not cover all aspects of interest [30] Data richness ⮚ Potentially small sample sizes with unequal representation [10] ⮚ Limited transferability of findings, due to localized data collection, purposive sampling and participant self-selection [30] Cost-effectiveness ⮚ Not all the data collected can always be included in the data analysis [10]
Engagement and creating	Participatory action research and CBPR*	Participatory action research Involvement ⮚ Equal power relations between researcher and researched [9] ⮚ Ethical form of research [9] Data richness ⮚ Addressing problems, generating knowledge and finding solutions together [9] ⮚ Citizens open up more, resulting in in depth and rich data that are substantial and emotionally nuanced [9] CBPR* Involvement ⮚ Applicable research findings can be put into practice in specific communities directly [13] Data richness ⮚ A deep understanding of a community’s unique circumstances [25,33,34] ⮚ The unique perspective of citizens on the research problem and process [13]	Participatory action research Involvement ⮚ Difficulty establishing a genuinely collaborative project [9] ⮚ Lack of common ground between research and practice [9] ⮚ Unrealistic citizen expectations about the research [9] ⮚ Time-consuming and expensive [9] ⮚ Receiving funding can be difficult [9] Data richness ⮚ Potentially unrepresentative samples [9] CBPR Involvement ⮚ Inconsistent attendance by citizens [25] ⮚ Long recruitment periods [25] Data richness ⮚ Small sample sizes [25] ⮚ Participants experiencing fatigue [25] Data quality ⮚ Lower research validity and reliability [13] ⮚ Less elaborate survey instruments [13] ⮚ Changes in program components [25] ⮚ Less control over data collection [13]

**Note.** All advantages and disadvantages are mentioned in mainly food behaviour studies and we allocated them to one of the four areas that we mapped: cost-effectiveness, involvement, data richness and data quality; *CBPR = community-based participatory research
